# Supplementary material for: How Did Breast Cancer Patients Fare during Different Phases of the COVID-19 Pandemic in Norway Compared to Age-Matched Controls?
Source: Cancers (Basel). 2024 Jan 31;16(3):602. doi: 10.3390/cancers16030602 (PMC10854821; doi:10.3390/cancers16030602)
Supplement: Supplementary file 1 [file cancers-16-00602-s001.zip › cancers-2813084-supplementary.pdf]

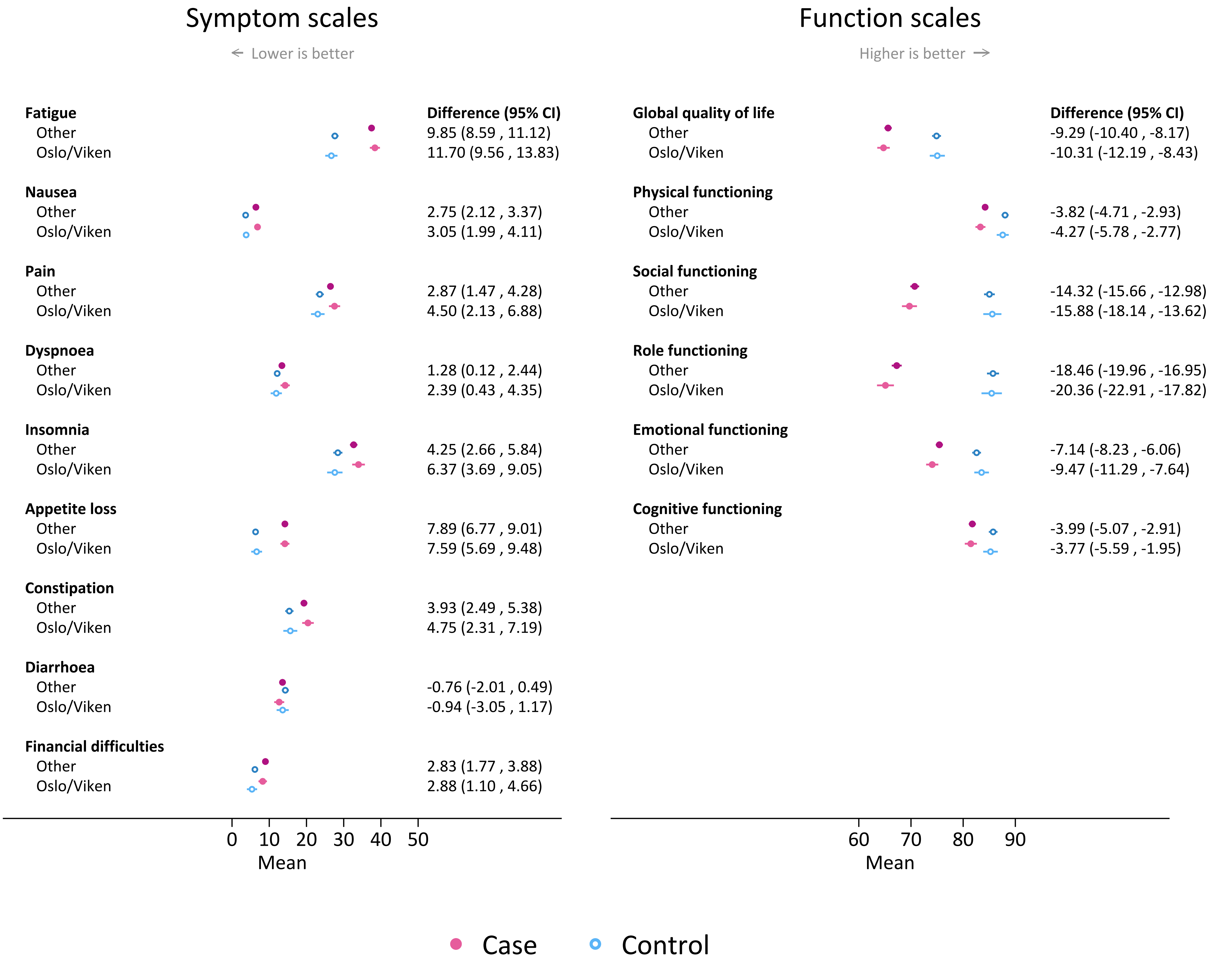
Symptom scales
← Lower is better
Function scales
Higher is better →
Fatigue
Other
Oslo/Viken
Difference (95% CI)
9.85 (8.59 , 11.12)
11.70 (9.56 , 13.83)
Nausea
Other
Oslo/Viken
2.75 (2.12 , 3.37)
3.05 (1.99 , 4.11)
Pain
Other
Oslo/Viken
2.87 (1.47 , 4.28)
4.50 (2.13 , 6.88)
Dyspnoea
Other
Oslo/Viken
1.28 (0.12 , 2.44)
2.39 (0.43 , 4.35)
Insomnia
Other
Oslo/Viken
4.25 (2.66 , 5.84)
6.37 (3.69 , 9.05)
Appetite loss
Other
Oslo/Viken
7.89 (6.77 , 9.01)
7.59 (5.69 , 9.48)
Constipation
Other
Oslo/Viken
3.93 (2.49 , 5.38)
4.75 (2.31 , 7.19)
Diarrhoea
Other
Oslo/Viken
-0.76 (-2.01 , 0.49)
-0.94 (-3.05 , 1.17)
Financial difficulties
Other
Oslo/Viken
2.83 (1.77 , 3.88)
2.88 (1.10 , 4.66)
0 10 20 30 40 50
Mean
Global quality of life
Other
Oslo/Viken
Difference (95% CI)
-9.29 (-10.40 , -8.17)
-10.31 (-12.19 , -8.43)
Physical functioning
Other
Oslo/Viken
-3.82 (-4.71 , -2.93)
-4.27 (-5.78 , -2.77)
Social functioning
Other
Oslo/Viken
-14.32 (-15.66 , -12.98)
-15.88 (-18.14 , -13.62)
Role functioning
Other
Oslo/Viken
-18.46 (-19.96 , -16.95)
-20.36 (-22.91 , -17.82)
Emotional functioning
Other
Oslo/Viken
-7.14 (-8.23 , -6.06)
-9.47 (-11.29 , -7.64)
Cognitive functioning
Other
Oslo/Viken
-3.99 (-5.07 , -2.91)
-3.77 (-5.59 , -1.95)
60 70 80 90
Mean
Case
Control
